# Using the Behavioral Risk Factor Surveillance System (BRFSS) for Exposure Tracking: Experiences from Washington State

**DOI:** 10.1289/ehp.7148

**Published:** 2004-08-03

**Authors:** Denise M. Laflamme, James A. VanDerslice

**Affiliations:** Office of Environmental Health Assessments, Washington State Department of Health, Olympia, Washington, USA

**Keywords:** BRFSS, environmental health, environmental public health tracking, exposure assessment

## Abstract

One of the goals of the National Environmental Public Health Tracking Network is to link environmental data with chronic disease data as a means of improving our understanding of the environmental determinants of disease. Such efforts will rely on the ongoing collection of population exposure information, and there are few systems in place to track population exposures. In many cases, exposures can be estimated by combining environmental contaminant data with data about human behaviors. The Behavioral Risk Factor Surveillance System (BRFSS) provides a good opportunity to implement tracking of exposure-related behaviors. Washington State has used the BRFSS to collection information on environmentally related knowledge, attitudes, and behaviors. In this article we present case studies of modules covering drinking water, perceptions of environmental risk, and radon awareness and testing. Data on exposure-related behaviors have been useful for population exposure assessments and program evaluation. Questions about knowledge and attitudes and perceptions of environmental issues were not as useful because they lacked sufficient detail from which to modify existing education efforts. In some cases these data had not been used at all, indicating that the need for the data had not been well established. National development efforts should focus on compiling existing questions and developing questions on topics that are a priority at the state and national levels to be included as core questions and optional modules in future BRFSS surveys.

One of the primary goals of the National Environmental Public Health Tracking Network (EPHTN) is the development of the methods and data systems to link environmental data with chronic disease data in order to improve our understanding of the environmental determinants of disease [Centers for Disease Control and Prevention (CDC) 2004c]. Understanding exposure patterns in a population is a key element in linking environmental contamination to health outcomes. Although many studies have measured or estimated exposures in a defined population, there are few ongoing, systematic data collection efforts designed to track population exposures, particularly at the state or local level (CDC 2003b; Schober et al. 2003).

Many exposures are strongly influenced by behavior, including the types, frequencies, and amounts of foods and water consumed; the time spent and level of activity while breathing in different indoor environments; the time spent and level of hand-to-mouth activity in children; and the frequency of hand washing (Wallace et al. 1989; Yang et al. 1998). Individual efforts to test private wells or radon levels in the home and to take actions to reduce contaminant levels in one’s immediate environment are examples of individual behaviors that influence exposure by changing contaminant levels in their immediate environment.

In addition to the behaviors themselves, it is important to have a good understanding of the knowledge and attitudes of the individuals because these underlie the resulting behaviors. Given the importance of knowledge, attitudes, and behavior (KABs) as determinants of exposure, current data systems designed to capture information about KABs may be valuable tools for developing ongoing population exposure tracking efforts. One of the most widely developed systems is the Behavioral Risk Factor Surveillance System (BRFSS), an ongoing state-based telephone survey of randomly selected noninstitutionalized adults. The BRFSS is sponsored by CDC and is conducted in all 50 states, the District of Columbia, Puerto Rico, the Virgin Islands, and Guam (CDC 2004a). Survey participants are at least 18 years of age, live in the United States, and speak English (some states include the BRFSS in Spanish).

The survey instrument consists of a core set of questions developed by CDC that are used in all locations, optional modules of questions also developed by CDC that cover specific topic areas that may be used by a state, and questions developed and added by states. CDC’s optional modules of questions are available for state health departments to include based on state data needs and availability of funds to pay for them. In addition, states and local municipalities can develop their own modules of questions to include on the survey for their area.

The BRFSS primarily collects data on chronic diseases, injuries, infectious illnesses, and the behavioral factors underlying these conditions (Figgs et al. 2000). For many of these topics, BRFSS is the main source of state-level prevalence information, and BRFSS data are routinely used to set and track national health objectives such as Healthy People 2010 (Mokdad et al. 2003).

Many states have included environmental health–related questions on the BRFSS; however, most of these questions have been developed at the state level to address specific issues for that state. The heterogeneity of these issues is evidenced by the wide range of topics addressed, including asbestos, drinking water testing, food handling and safety, hanta-virus, lead, Lyme disease, rabies, and West Nile virus.

The Washington State Department of Health (WA DOH) has used the BRFSS to collect data on a variety of environmental health topics since 1990. Previously, there had been no analyses of how successful these questions were in addressing data needs within our agency or any assessment of how programs within the agency had used the results. The purpose of the present study was to compile and examine all environmental health data gathered in Washington using the BRFSS, assess the use and usefulness of these data, and examine the types of information gathered through the BRFSS that may have the greatest utility for ongoing exposure tracking efforts.

## Materials and Methods

We collected and compiled Washington’s BRFSS instruments and data sets and reviewed them to identify questions pertaining to environmental health. We then generated weighted frequencies and/or summary statistics using STATA (version 7.0; Stata Corporation, College Station, TX). Data were weighted to account for different probabilities of selection of each household and the number of adults in each household and to account for differences in the surveyed population compared with the general population. Some questions asked about characteristics of the household instead of the respondent. For those questions, analyses were weighted to adjust for the probability of selection of the household only. For some results, the percentage of responses does not add up to 100% because of respondents who reported they did not know or who refused to answer some questions. In all cases the percentages reported are based on weighted proportions and thus are estimates of the proportion of the entire population having that characteristic.

To evaluate the use and usefulness of the environmental health data collected using the BRFSS, we met with program managers from each program in the Division of Environmental Health as well as program managers from other assessment units in WA DOH to ascertain if the data had been used, how the results influenced policy or programmatic decisions, and how useful the data were in helping the program managers in developing new approaches or in evaluating their current programs. The compiled BRFSS results were also presented to senior managers at the Division of Environmental Health in order to obtain their input on the usefulness of the existing data and to identify what data they needed to more effectively run their programs. This process was conducted as part of the development and production of a comprehensive document addressing health indicators in the state (WA DOH 2002). A main goal of this exercise was to help plan for and critically evaluate future BRFSS environmental health questions.

## Results

From 1990 through 2004 WA DOH incorporated 13 environmental health–related modules into the BRFSS (Table 1). Environmental health–related modules were included in 9 of the 15 years. The statewide sample size ranged from 2,101 in 1990 to 4,826 in 2002.

The following sections summarize the BRFSS results and data use on three of these topic areas: drinking water, perceptions of environmental problems, and radon. These topic areas were chosen to illustrate different types of questions and the variability in how WA DOH programs used the results. The compiled results from all the environmental health questions are available elsewhere (WA DOH 2004a).

### Drinking water.

Between 1996 and 2000, Washington State included three modules of questions regarding drinking water. These questions covered exposure-related behaviors (e.g., source of drinking water, testing of private wells, and treatment of tap water) and attitudes (e.g., reasons for using bottled water or water filters).

Most households reported receiving their drinking water from city water systems. This proportion increased from 68 to 77% between 1996 and 2000 (Table 2) and appeared to have been offset by a reduction in the proportion of households using private wells, which decreased from 17 to 10% over the same time period.

In 1996, 76% of households using private wells reported having their well tested at some point (Table 3). This percentage increased to 83% for 1998. About two-thirds reported testing their well within the last 3 years in both the 1996 and 1998 surveys (data not shown). The percentage of people who reported they did not know when their well had been tested decreased from 7 to 2% between 1996 and 1998. Of the households who reported testing their well, 6% recalled that the tests indicated some type of contamination.

In 2000 most households (83%) reported getting their drinking water from the tap, with the remainder reporting using bottled water or water from a water cooler as their usual source of drinking water (Table 4). Less than 1% reported using some “other source.” Forty percent of households using tap water reported using a water filter (Table 4). When asked why they used a water filter or bottled water, 37% responded that it was because of the water’s appearance, taste, or smell; 19% responded that it was because they were concerned that their water was unsafe; and 33% said it was for both of these reasons.

#### Uses of data.

Results concerning household water supply have provided the only reliable source of information on the number of people using private wells in the state. Previous estimates were derived by summing the reported number of service connections by all public water supplies for the total number of households in the state. The data from the BRFSS were used to revise substantially the previous estimates of the number of households on private wells (WA DOH 2002).

The responses to questions from households using private wells, combined with responses to behavior questions regarding the type of water used for drinking and the use of water filters, have been used to estimate population exposures to bacterial contamination and nitrate among private well owners. These behavior data along with the attitude data about why bottled water or water filters were used have been used in training water utility operators about consumer perceptions of water quality in public water supplies. Finally, behavior data on private well testing have been provided to local public health authorities to help guide their efforts in managing private well-water quality.

### Perceptions of environmental problems.

In 1995 Washington State included questions on BRFSS to gauge public perceptions about the importance of various environmental issues. These questions were derived from those developed by the Northeast Tri-County Health District, which consists of Ferry, Stevens, and Pend Oreille counties in northeastern Washington State, and used in their 1994 county-level BRFSS (Gilmore Research Group 1995). For each environmental issue, the respondent was asked if it was “a problem in your community” and allowed to respond “yes,” “yes somewhat,” or “no.” The environmental issues were indoor and outdoor air quality, drinking water, workplace hazards, solid waste, pesticide use, wastewater, and hazardous waste sites.

Outdoor air quality was the issue most frequently identified as a problem in the community, with 22% responding that it was a problem or somewhat of a problem (Table 5). Indoor air quality was perceived to be a problem by only 6% of the respondents. For the other issues, about 10–15% of the respondents thought they were at least somewhat of a problem. Just more than half the respondents (54%) did not think that any of the environmental issues were a problem in their community.

#### Uses of data.

We could find no documentation of the rationale for including these questions on the statewide BRFSS. Environmental health program managers at WA DOH did not recall using these data and did not feel that the results of these questions provided useful information for program activities or public outreach.

Although these data have not been used at the state level, they have been used at the county level to help set priorities. The Northeast Tri-County Health District used these results as part of a comprehensive assessment of environmental hazards for the three-county area (Gilmore Research Group 1995). In 1996 similar perception questions were included on the BRFSS for Clark and Snohomish counties (Snohomish Health District 1997; Southwest Washington Health District 1997). The results of these perception questions were used in conjunction with local environmental data and stakeholder input to set health priorities and to guide public health planning efforts at the local level.

### Radon.

Several behavior and knowledge questions about radon were included on the 1990, 1993, and 1997 BRFSS’s. These questions were part of an optional module developed by CDC. The intent of this module for Washington State was to gather information on the impact of efforts to educate the public about the risks of radon.

The proportion of the population that had heard of radon gas increased slightly from 72% in 1990 to 77% in 1993 (Table 6). In 1990, 81% of the respondents agreed with the statement that radon gas was harmful to health (data not shown). However, there was a steady decline in the proportion agreeing and a corresponding increase in the proportion answering “don’t know,” indicating an erosion in public awareness about radon (15% in 1990 to 27% in 1997). Less than one-third reported that they knew how to test for radon.

The percentage of households tested for radon gas was relatively low and did not change over time, ranging from 7 to 9% between 1990 and 1997 (Table 6). The percentage of households planning to test for radon gas was also low, staying between 6 and 8% during this time. The percentage of households planning to test for radon gas was even lower in households never tested for radon (4–6%).

#### Uses of data.

The questions in the radon module provide information about awareness, knowledge, and protective behaviors regarding radon. Program managers felt that information about behaviors as well as knowledge was more useful for program planning and evaluation than information from knowledge or attitude questions alone. Early results were used to guide and evaluate WA DOH’s radon awareness program; however, that program was discontinued in 1994. Data on household testing for radon have been used in a recent state compilation of environmental health problems (WA DOH 2002) and to help set priorities for Washington State’s Comprehensive Cancer Control Plan (WA DOH 2004b).

## Discussion

CDC (2004c) defines environmental public health tracking as

the ongoing collection, integration, analysis, and interpretation of data about environmental hazards, exposure to environmental hazards, and human health effects potentially related to exposure to environmental hazards.

In this context, “environmental hazards” refers to chemical, radiologic, or biologic agents in the environmental that, because of their inherent characteristics, may pose a risk to people who are exposed. Operationally, data about environmental hazards include measures of the levels of these agents in environmental media, rates, or amounts of agents released into the environment and estimated environmental concentrations or emissions derived from modeling.

Although environmental monitoring and disease surveillance systems are well established throughout the country, there are few working examples of systems collecting ongoing, systematic data about environmental exposures. Such systems are the cornerstone of efforts to link environmental data to health data.

The BRFSS provides perhaps the best opportunity for a national systematic collection of data on behavioral determinants of exposure as well as the knowledge, perceptions, and attitudes underlying these behaviors. The marginal cost of adding questions to this established program is much less than the cost of designing and implementing a new survey. The sampling design and protocols are well established, the administrative mechanisms are in place, and there are a number of contractors who have experience using this survey in the field.

Perhaps the most attractive feature of BRFSS is its design, which allows individual states to tailor the content of the survey to meet state needs through the use of state-added questions and the optional use of CDC-developed modules. In addition, states or localities can increase the sample size overall or selectively target specific groups to meet analytical objectives. Consequently, prevalence estimates can be derived for each state, and for localities within states, with appropriate standard errors through the use of weights. This is in contrast to the other available national health surveys such as the National Health and Nutrition Examination Survey (NHANES) and the National Health Interview Survey, which are designed to provide national-level prevalence estimates.

One alternative is the development of surveys similar to the NHANES that could be conducted at the state or regional level to generate results for specific states or geographic regions. Such surveys could incorporate the use of biomarkers similar to the Second National Report on Human Exposure to Environmental Chemicals (CDC 2003b) to provide distributions of body burdens for state or regional populations. However, only New York City has developed and deployed such a survey, and sources of funding for a national or multistate effort to develop and conduct a state-level health and nutrition examination survey have yet to be identified.

The constant effort needed to collect exposure-related behavior information over time will require an ongoing institutional demand or mandate. Most environmental data are collected because it is required by federal or state legislation as part of regulatory activities. Disease surveillance systems were developed originally to meet a very real need to control outbreaks of communicable diseases, and this need was codified into rules and regulations covering notifiable conditions at the state level. Although an EPHTN would require ongoing, systematic data on a variety of environmental exposures, at present there are few examples of regulations or laws that require such information be collected. The first environmental health BRFSS core questions to be developed by CDC asked whether the respondent had experienced an illness due to environmental factors; these questions were part of the 2004 BRFSS and are currently included in the draft 2005 survey instrument. CDC has also developed optional modules on radon, environmental tobacco smoke, indoor air quality, and the home environment. The value of collecting such information needs to be clearly demonstrated to institutionalize these data collection efforts.

Standardizing questions across states and increasing the dissemination to a national audience will enhance the value of these data. Data from environmental health–related BRFSS questions have only rarely been published in the scientific literature (CDC 2003a; Kreutzer et al. 1999), and many of the existing results are found exclusively in state health department reports that are not abstracted by the major search services. A list of questions used in the core and optional modules is available on the CDC web site (CDC 2004b); however, there is no comprehensive database of state-added environmental health–related questions. Such a database would help states identify new BRFSS topics to consider and would provide a resource for program staff in states considering development of new BRFSS questions. Through the current EPHTN cooperative agreements, Washington State will be developing a repository of environmental health questions that have been used in the BRFSS, to share specific questions, results, and information about the validity of the available questions.

Within WA DOH there has not been a consistent approach for recommending and developing environmental health questions. Because of this, it has been difficult to determine how well some of the BRFSS questions met program needs within the agency or how some of the results were used. The information needs of the organization may not have been adequately developed and/or communicated to the staff designing the BRFSS module.

For many of the older questions (e.g., questions from the early 1990s), it was difficult to determine the program need that the questions were meant to address and how questions had been developed. In some cases it was difficult to ascertain how or if the WA DOH programs had used results of BRFSS questions they had requested because the program staff had changed and there was little or no documentation to show that the data had been analyzed or used. It appeared that the lack of use may have been because of changes in staff between the time the questions were proposed and when the results were available, or because of a lack of personnel with the skills to access and correctly analyze these data.

Even if the information needs were well conceived and communicated, the questions developed for the module may not have generated the type of data needed to address the information needs. For example, the environmental perception results did not accurately reflect actual known risks, indicating a need for better risk communication: Ambient air quality was identified as an environmental problem by a much larger proportion of respondents than indoor air quality (22.3 vs. 5.2%; Table 5). However, exposure studies have identified indoor air as the main source of exposure to many air pollutants and a source of some of the highest noncancer and cancer risks (U.S. Environmental Protection Agency 1990; Wallace et al. 1987). While knowing that such knowledge gaps exist is important, most questions addressing knowledge and attitude were generally too broad to provide sufficient detail from which to base modifications of existing educational materials.

Cognitive testing, pretesting, and studies of question validity are essential for ensuring that questions generate meaningful information (Aday 1996). Finally, the need for the information may not have been great enough to have managers take the time and resources to access, analyze, and incorporate the results into their programs, or the information needs may have changed during the 1.5–2 years that elapse between deciding to use the BRFSS and receiving the final data set from CDC.

The use of BRFSS does have clear limitations. WA DOH programs are charged $850 for each state-added question included in the BRFSS. While this cost has been a barrier for some programs, the overall length of the survey has become a more important constraint. Because of concerns about the declining response rate, WA DOH decided to limit the total length of the survey to 25 min. Given the length of the core survey, usually < 12 min are available for all optional modules and state-added questions. This constraint needs to be managed to avoid competition between state health department programs wishing to use the BRFSS.

In response to an increase in interest in using the BRFSS, our process for selecting state-added questions to be added to the survey has been modified over the last 5 years to require explicit descriptions of the information needs, how the data will be analyzed to meet these needs, and who will be responsible for conducting the data analysis. These factors, as well as evidence that previously collected data have actually been used by WA DOH programs, are used as criteria in the selection of questions to be included.

From our experience in Washington State, many environmental health professionals do not see the value in using tools such as the BRFSS to monitor KABs that lead to environmental exposures. We have observed this among WA DOH managers as well as among environmental health directors from local health jurisdictions around the state. This may be due in part to the nature of traditional environmental public health functions, which have centered on developing and enforcing standards in areas such as food safety, drinking water safety, radiation protection, and solid waste disposal.

Understanding environmentally related KABs will likely become more important as environmental health professionals begin to face issues such as large-scale polychlorinated biphenyl and arsenic contamination. In these circumstances, risk management cannot focus on regulating releases or mandating environmental remediation but rather must rely on efforts of health promotion programs to educate and motivate individuals to take appropriate steps to minimize their exposures. Tools such as the BRFSS will be critical for designing and evaluating such efforts.

## Conclusions

The BRFSS offers an excellent opportunity to implement a system for tracking important exposure-related behaviors as part of the EPHTN. The relatively low marginal cost of adding nationally developed optional modules or state-added questions, the flexibility inherent in the sample design, and the well-developed infrastructure and procedures make the BRFSS an attractive option for exposure tracking. Although environmental health topics have not typically been included in the BRFSS at the national level, several states have developed and successfully used the BRFSS to collect data about exposure-related behaviors and the knowledge and attitudes that underlie these behaviors. As with any survey, there are limitations to the accuracy of data recall. Even so, such data on past behaviors have been useful for population exposure assessments and program evaluation. Questions about perceptions of environmental problems alone have not been seen as useful because they have lacked sufficient detail from which to modify existing education efforts. National development efforts should focus on compiling existing questions and experiences and identifying topics that are a priority at the state and national levels to be included as core questions and optional modules in future BRFSS surveys.

## Figures and Tables

**Table 1:** Environmental health–related modules included on Washington State BRFSS and use of data at the state level, 1990–2004.

Topic	Year	Data used?
Drinking water source, well testing	1995, 1996, 1998, 2000	Yes
Environmental tobacco smoke*^a^*	2000	Yes
Fish consumption, levels and awareness of fish advisories	2002, 2004	Yes
Hazardous waste sites, perception of problem	1995	No
Household heating source	1996	No
Household mold presence	2004	NA
Indoor air quality, perception of problem	1995, 1996	No
Outdoor air quality, perception of problem	1995, 1996	No
Illnesses perceived to be caused by indoor and outdoor air contamination*^b^*	2004	NA
Pesticides, household use and perception of problem	1995, 2000	Yes
Radon awareness and testing behaviors*^a^*	1990, 1993, 1997	Yes
Waste water and solid waste disposal, perception of problem	1995, 1996	No
Water recreation, frequency of use	1990	No
West Nile virus, awareness and protective behaviors	2004	NA
Workplace hazards, perception of problem	1995	No

NA, not applicable.

a CDC optional module.

b CDC core question for 2004.

**Table 2:** Source of household drinking water, Washington State, 1996–2000.

	1996		1998		2000*^a^*	
Question	No.	% (95% CI)	No.	% (95% CI)	No.	% (95% CI)
City water system	2,519	68.4 (66.6–70.2)	2,627	72.3 (70.5–74.1)	2,757	76.9 (75.5–78.3)
Small community system	215	6.0 (5.0–7.0)	248	7.1 (6.1–8.1)	235	6.6 (5.8–7.4)
Private well	545	16.5 (15.1–17.9)	472	13.7 (12.3–15.1)	368	10.0 (9.0–11.0)
Other	80	2.2 (1.6–2.8)	59	1.6 (1.2–2.0)	65	1.9 (1.5–2.3)
Don’t know	191	5.5 (4.5–6.5)	155	4.4 (3.4–5.4)	140	4.1 (3.3–4.9)

95% CI, 95% confidence interval.

a The wording of the drinking water source question for 2000 was modified from the question used in 1996 and 1998. Questions in 1996 and 1998: “What is the source of your home’s drinking water? Does it come from: a city or district supply, a community system, a private well, or some other source?” Questions in 2000: “Where does the water for your household come from? A private well serving just your household, a community well or other small water system which serves fewer than 15 homes, a city or municipal water supply, other?”

**Table 3:** Private domestic water well testing, Washington State, 1996 and 1998.

	1996		1998	
Question	No.	% (95% CI)	No.	% (95% CI)
Has your well water ever been tested?				
Yes	420	75.5 (71.0–80.0)	395	82.7 (78.4–87.0)
No	75	13.1 (9.8–16.4)	38	8.8 (5.5–12.1)
Don’t know	50	11.4 (7.7–15.1)	39	8.4 (5.5–11.3)
Did the results from well testing indicate the presence of any contaminants?				
Yes	25	5.9 (3.4–8.4)	27	6.1 (3.6–8.6)
No	371	89.1 (85.8–92.4)	357	91.5 (88.6–94.4)
Don’t know	23	4.8 (2.6–7.0)	11	2.3 (0.7–3.9)

**Table 4:** Drinking-water source, use of water filters among users of tap water, and reasons for water-filter use, Washington State, 2000.

Question	No.	% (95% CI)
Where do you usually get the water that you drink at home?		
Tap	2,962	83.0 (80.5–85.5)
Bottled water or from water cooler	572	15.7 (13.3–18.1)
Other source	25	0.7 (0.1–1.3)
Do you use a water filter for your household drinking water? (tap water users only)		
Yes	1,188	39.7 (37.9–41.5)
No	1,762	59.8 (58.0–61.6)
What is the main reason that you use a water filter or bottled water for your drinking water at home?		
Don’t like the way the water looks, tastes, or smells	643	37.0 (34.6–39.4)
Concerned that the water is not safe to drink	332	18.8 (16.8–20.8)
Both of these two reasons	588	33.3 (30.9–35.7)
Some other reason	170	9.4 (8.0–10.8)

**Table 5:** Opinions about environmental problems in the community, Washington State, 1995.

	Yes		Yes, somewhat		No	
Question*^a^*	No.	% (95% CI)	No.	% (95% CI)	No.	% (95% CI)
In your opinion, is (*topic is inserted*) a problem in your community?						
Outdoor air quality?	458	13.3 (12.1–14.5)	305	9.0 (8.0–10.0)	2,543	76.6 (73.5–79.7)
Drinking water quality?	376	11.1 (8.7–13.5)	150	4.4 (2.8–6.0)	2,742	82.3 (79.2–85.0)
Hazards in your workplace?	207	9.5 (8.1–10.9)	85	4.1 (3.1–5.1)	1,842	85.5 (83.9–87.1)
Solid waste management?	258	7.3 (6.3–8.3)	82	2.7 (2.1–3.3)	2,942	88.2 (87.0–89.4)
Pesticide use and control?	253	7.1 (6.1–8.1)	98	2.8 (2.2–3.4)	2,790	84.3 (85.4–85.7)
Wastewater management?	232	7.0 (6.0–7.0)	77	2.3 (1.7–2.9)	2,895	86.8 (85.6–88.0)
Hazardous waste sites?	216	6.3 (5.5–7.1)	48	1.6 (1.0–2.2)	2,942	86.8 (85.6–88.0)
Air quality inside your home?	87	2.6 (2.0–3.2)	83	2.6 (2.0–3.2)	3,138	93.6 (92.6–94.6)

a Introductory statement: “These questions ask about the quality of the environment in your community. I’m going to read you a list of items and for each item I’d like you to tell me if, in your opinion, it is a problem in your community.

**Table 6:** Radon awareness and radon testing, Washington State, 1990, 1993, and 1997.

	1990		1993		1997	
Question	No.	% (95% CI)	No.	% (95% CI)	No.	% (95% CI)
Have you heard of radon, which is a radioactive gas that occurs in nature?						
Yes	1,522	71.9 (69.7–74.1)	1,996	76.5 (74.7–78.3)		
No	539	26.4 (24.3–28.4)	568	22.6 (20.8–24.4)	Not asked						
Don’t know	40	1.7 (1.1–2.3)	22	0.9 (0.5–1.3)		
Refused	0	NA	0	NA		
Do you know how to test your home for the presence of radon gas?						
Yes	428	28.6 (26.2–31.0)	515	24.9 (22.9–26.9)		
No	1,034	67.3 (64.7–69.9)	1,456	72.7 (70.6–74.8)	Not asked						
Don’t know	60	4.1 (3.0–5.2)	45	2.3 (1.6–3.0)		
Refused	0	NA	2	0.1 (0–0.3)		
Has your household air been tested for the presence of radon gas?						
Yes	112	6.8 (5.4–8.2)	164	8.7 (7.3–10.1)	273	8.3 (7.1–9.5)
No	1,346	89.0 (87.2–90.8)	1,753	86.5 (84.9–88.1)	3,030	83.5 (82.0–85.0)
Don’t know	64	4.2 (2.9–5.5)	98	4.7 (3.7–5.7)	285	7.7 (6.7–8.7)
Refused	0	NA	3	0.2 (0.02–0.4)	16	0.4 (0.1–0.7)
Do you, or does anyone in your home plan to have your household air tested for radon within the next year?						
Yes	101	6.8 (5.2–8.4)	138	7.8 (5.9–9.8)	198	6.1 (5.1–7.1)
No	1,280	84.3 (82.1–86.5)	1,708	83.4 (81.6–85.2)	3,090	84.9 (83.5–86.3)
Don’t know	141	8.9 (7.2–10.6)	170	8.9 (7.4–10.3)	300	8.5 (7.4–9.6)
Refused	0	NA	2	0.1 (0–0.3)	16	0.5 (0.2–0.8)

NA, not applicable.
